# Methodological approach for an integrated female-specific study of anxiety and smoking comorbidity

**DOI:** 10.3389/fpsyt.2023.1267753

**Published:** 2023-11-24

**Authors:** Samantha G. Farris, Jacqueline E. Smith, Dana R. Steinberg, Brianna R. Altman, Geralyn M. Lambert-Messerlian, Shira I. Dunsiger, David M. Williams, Michael E. Saladin, Ana M. Abrantes

**Affiliations:** ^1^Department of Psychology, Rutgers, The State University of New Jersey, New Brunswick, NJ, United States; ^2^Pathology and Laboratory Medicine and Obstetrics and Gynecology, Alpert Medical School of Brown University, Providence, RI, United States; ^3^Department of Behavioral and Social Sciences, Brown University School of Public Health, Providence, RI, United States; ^4^Department of Psychiatry and Human Behavior, Alpert Medical School of Brown University, Providence, RI, United States; ^5^Department of Health Science and Research, Medical University of South Carolina, Charleston, SC, United States; ^6^Behavioral Medicine and Addictions Research Unit, Butler Hospital, Providence, RI, United States

**Keywords:** menstrual cycle, progesterone, estradiol, anxiety, nicotine reinforcement, ecological momentary assessment

## Abstract

Two primary ovarian hormones that fluctuate across the female menstrual cycle—estradiol and progesterone—have been independently linked in separate literatures to nicotine reinforcement and anxiety psychopathology. We identify existing methodological limitations in these literatures, describe an example protocol that was developed to address such limitations, highlight case examples, and offer insights on the resulting advantages and challenges. This protocol was an observational, prospective, within-subjects study of female cigarette smokers who were followed over the course of a complete menstrual cycle. Non-treatment seeking, female cigarette smokers (*N* = 50), between the ages of 18–40 who have a normal menstrual cycle (25–35 days in length) were recruited from the community. Females with anxiety or mood psychopathology represented 38.0% of the sample. Salivary progesterone and estradiol were assessed each morning via at-home saliva collection methods. Self-reported within-day momentary ratings of anxiety and nicotine reinforcement were collected using ecological momentary assessment (EMA) via a mobile app. Protocol compliance was >85%. Within- and between-subjects heterogeneity was observed in the progesterone and estradiol, anxiety, and nicotine craving measures, especially in the context of anxiety psychopathology. We aimed to integrate the anxiety and nicotine dependence literatures and advance the empirical study of the role of ovarian hormones. This protocol reflects an intensive, yet feasible approach to collecting daily-level naturalistic data related to estradiol, progesterone, anxiety, and nicotine reinforcement.

## Introduction

Cigarette smokers are approximately twice as likely to have an anxiety disorder compared to the general population (1–3), and anxiety is associated with poorer cessation outcomes (2, 4, 5). Females are twice as likely to have an anxiety or related stress disorder compared to males (6). While the prevalence of smoking is higher in males relative to females (7), females are at greater risk for tobacco-related disease (8, 9) and are less successful in quitting smoking (10–15), perhaps because they experience greater reinforcing effects from cigarettes (i.e., nicotine reinforcement). Compared to males, females are more sensitive to the rewarding and stimulating effects of nicotine (16, 17). Females also report greater smoking for stress-relief than males (18), which may increase the (negative) reinforcing value of cigarettes (19). For example, following negative-mood induction, females (vs. males) demonstrate greater nicotine reinforcement: they report stronger craving (20), initiate smoking more quickly (21), consume more nicotine (22), and report greater cigarette “liking” (22). Identifying female-specific mechanisms underlying the anxiety-nicotine reinforcement link has the potential to address sex and mental health disparities in cessation outcomes.

Ovarian hormones, particularly estradiol and progesterone, which fluctuate throughout the menstrual cycle, have been *independently* examined as female-specific etiological factors in anxiety (23) and in cigarette smoking (24). Indeed, these research literatures have remained siloed which has limited our ability to study how ovarian hormones influence the *interplay* between anxiety and cigarette smoking. Our group has conducted initial work that is explicitly aimed at integrating these literatures, and in turn, our understanding of female-specific biological influences on anxiety and smoking. In this article, we present an initial overview of how estradiol and progesterone typically fluctuate during the menstrual cycle, and then briefly review the key literature that has been published on ovarian hormone influences in anxiety and nicotine reinforcement. Further, we discuss methodological drawbacks of the extant literature and introduce our novel experimental design to address those limitations. We provide illustrative case examples that highlight the inter- and intra-individual variability in hormonal fluctuations, anxiety, and craving. We conclude with a discussion of limitations and methodological considerations to inform future empirical inquiry.

### Estradiol and progesterone during the menstrual cycle

The menstrual cycle can be characterized by phases that reflect changes in fluctuating levels of estradiol and progesterone (25, 26). The onset of menses (blood flow) marks both the beginning of the menstrual cycle and the follicular phase. During the follicular phase, progesterone is very low, whereas estradiol is low during the early phase, and increases as the follicle develops, peaking sharply in the late follicular phase prior to ovulation. The increase in estradiol causes a surge of luteinizing hormone (LH), which signals ovulation and formation of the corpus luteum, a temporary organ that primarily produces progesterone (26). The luteal phase begins with rising levels of progesterone and estradiol, with progesterone reaching its peak at the midpoint of the phase and estradiol reaching a second peak. This is followed by a late-phase, rapid, large decrease in progesterone and moderate decrease in estradiol. The median menstrual cycle length is 28 days, with a normal range between 25 and 35 days (26). Most variability occurs in the follicular phase depending on when the follicle begins to develop whereas the luteal-phase duration is more constant (lasting 10–16 days) (26).

Fluctuations in estradiol and progesterone during the menstrual cycle likely influences smoking and anxiety (and their comorbidity) through various neurobiological processes implicated in drug reinforcement (via dopamine activation) (27), which has received most attention in the addiction literature, and emotion dysregulation (via serotonin, hypothalamic pituitary adrenal (HPA) axis, gamma-hydroxy-butyric acid (GABA) (28, 29)), which has received attention in the anxiety psychopathology literature. These are described in more detail below.

### Ovarian hormones and anxiety psychopathology

Periods of hormonal change have been associated with changes in emotional distress symptoms in both non-clinical (30) and clinical (23) populations. Clinically, hormonal influences have been linked to the etiology of panic disorder (31–33), social anxiety disorder (34), generalized anxiety disorder (35, 36), and anxiety-related disorders including obsessive-compulsive disorder (OCD) (37, 38) posttraumatic stress disorder (PTSD) (39), and pre-menstrual dysphoric disorder (PMDD) (40). Estradiol and progesterone contribute to the regulation of serotonin and allopregnanolone (respectively), which can have anxiolytic effects that may promote adaptive responding to stress (29). Thus, decreasing/low levels of estradiol and progesterone can increase risk for heightened negative affect, anxious arousal, and poor emotion regulation due to decreased serotonergic and allopregnanolone availability (29), which in turn results in less effective HPA axis regulation and reduced GABA activity (28, 29). Indeed, females report worsening of anxious arousal (39, 41), heightened startle and slower recovery from acute stress-induction (23), and less effective use of coping strategies (42) during the late-luteal/menstruation phase, where estradiol and progesterone are decreased, relative to other phases. Ovarian hormones may also play a role in fear extinction, thus influencing responses to certain anxiety treatments like exposure-based therapy (29). For instance, one study found that higher levels of progesterone on the day in which treatment was delivered (i.e., a single session of cognitive restructuring), was predictive of a larger response to treatment indicated by increased behavioral approach during a spider phobia task (43). Overall, these data underscore consistent links between ovarian hormones and affective experiences.

### Ovarian hormones and nicotine reinforcement

Hormonal fluctuations are further implicated in nicotine reinforcement and use behaviors. Broadly, across both preclinical and clinical studies, estradiol enhances while progesterone decreases nicotine’s reinforcing value (44). Preclinical data indicate that estradiol modulates the release of dopamine in the mesolimbic reward pathway (45) and increases dopamine release following nicotine administration (46). Drug reward and self-administration in animals is heightened when estradiol levels are high while progesterone levels are lower (27). More recent imaging data provide more evidence for the role of reward processing in the link between estradiol and smoking (47). For example, ventral striatal responsivity to smoking cues was found to be elevated during the estradiol-dominant menstrual phases, like the late follicular phase and mid-luteal phase, compared to phases characterized by low estradiol (47).

While heightened estradiol appears to exacerbate nicotine reinforcement, progesterone appears to attenuate smoking-related outcomes. Progesterone administration reduces drug reward and drug seeking behavior in female rats (27, 48–50). Progesterone administration (vs. placebo) during the follicular phase also attenuates the subjective positive reinforcing drug effects (51–53). Human data indicate that lower levels of progesterone in relation to estradiol are associated with greater puff intensity and puff number (54). Additionally, nicotine-deprived females who received intravenous nicotine reported lower positive subjective effects (e.g., “liking it”) when in the luteal (progesterone dominant) relative to the follicular (estradiol dominant) phase (55). As a result, studies have recommended that females may be more successful in quitting during the luteal phase (when progesterone is highest) (54, 56, 57), and have examined the use of exogenous progesterone to bolster cessation outcomes (58), though data supporting luteal phase protective factors are mixed due to inconsistencies in measurement, definition of cycle phase, and hormonal fluctuations within sub-phases.

### Implications for anxiety-smoking comorbidity

Despite the relevance of ovarian hormones to both anxiety and smoking reinforcement, there is a dearth of research examining how fluctuations of estradiol, progesterone, and progesterone’s metabolites throughout the menstrual cycle influence the *interplay* between anxiety and smoking reinforcement. Female smokers with anxiety may be particularly vulnerable to the emotional consequences of fluctuating ovarian hormones, resulting in maintained reliance on cigarettes. Of the limited literature, we are aware of one relevant population-based study that examines these relations. In a large representative sample of females (*N* = 11,648) between the ages of 18–55, the presence of past-month menstrual problems was associated with more frequent anxiety, depression, and fatigue, and those females with menstrual problems were more likely to smoke cigarettes (59). One additional relevant preliminary investigation conducted by our study team examined between-subjects differences (60) in female smokers (*N* = 23) with normal cycles not using hormonal birth control. Participants self-reported the first day of their last menstruation and completed cross-sectional self-report measures of emotion dysregulation and cigarette craving. Females who were assessed during the late-luteal phase (based on self-reported menses) reported higher levels of emotion regulation difficulties, relative to females who were assessed while in other menstrual phases (60). In addition, females who were assessed in the follicular phase reported higher levels of nicotine demand and craving relative to females assessed during other menstrual phases (60). These investigations provide preliminary evidence for the importance of considering anxiety and emotional factors more broadly in the context the menstrual cycle and smoking links.

### Methodological advancements

The above-mentioned preliminary literature is promising; however, it is limited in both content and methodological approach. Not only is there limited available literature on the *integration* of the anxiety and smoking literatures as it relates to the menstrual cycle, the disparate literatures in anxiety and smoking would each be bolstered by methodological advancements. Below we outline several common methodological limitations and advancement opportunities.

#### Limitation 1: exclusion of individuals with anxiety psychopathology from smoking studies

Smokers with psychopathology have been *systematically excluded* from the research on ovarian hormones and smoking. For example, of the 36 studies included in a meta-analysis on smoking and ovarian hormones (24), 30 studies excluded females with psychopathology or those who were receiving psychiatric treatment, four studies did not report any information, and only two studies considered psychopathology—one examined subclinical depression symptoms in female smokers without psychopathology (61) and another considered female smokers with a *history of* depression but excluded those with current depressive symptoms (62). In fact, depression is cited as a potential confounder condition that should be considered for exclusion in biobehavioral research focused on the menstrual cycle (25). While depression and anxiety can influence the menstrual cycle and influence reproductive hormones (63, 64), exclusion of individuals who smoke and have diagnosed psychopathology lacks ecological validity and representation of “real world” smokers.

#### Limitation 2: lack of direct measurement of ovarian hormones

The most common method for studying ovarian hormones is through estimation of the menstrual phase based on self-reported onset of menses and forward day counting (25). With this approach, menstrual phase is used as a proxy for endogenous progesterone and estradiol levels. However, day count shows only 50% agreement with menstrual phase when determined by serum progesterone and estradiol assay (65), likely in part due to recall bias and variability in cycle length. In a review of biobehavioral studies focused on the menstrual cycle, only 18% of these studies used saliva sampling and 34% used blood as an objective measurement of ovarian hormones (25). A recent review examining anxiety symptoms across the menstrual cycle found 8 of the 14 studies use self-report or day count alone for the assessment of cycle phase (66). Continued reliance on day count methods produces unreliable menstrual cycle phase determinations (66).

Direct measurement of estradiol and progesterone via analysis of blood or saliva samples is ideal, though there are several challenges. Normative ranges for defining menstrual phase are difficult to establish in blood serum and saliva due to the significant variability of hormone levels between people (25). While blood contains both bound and unbound hormones, saliva contains only unbound hormone levels—thus, the concentration of estradiol and progesterone is lower in saliva relative to blood. However, the advantage of saliva over blood measurement is that the concentration of hormones in saliva may better reflect the biologically available levels of these hormones (25). Another advantage of saliva sampling is that it offers a noninvasive, easily stored means to self-collect samples while mitigating the burden of in-laboratory blood draws required for serum (25). Thus, salivary sampling allows for more frequent and convenient sampling than other means of assessment and is particularly advantageous for studying the smoking-anxiety link.

#### Limitation 3: cross-sectional or static hormone assessments

Cross-sectional measurement of hormones can provide information about whether absolute hormone levels are related to an outcome of interest, but this approach offers limited insight on the dynamic fluctuations of estradiol and progesterone as a process-based mechanism underlying anxiety and craving. In the ovarian hormone and anxiety literature, in-lab paradigms are leveraged to look at specific associations between phase of the menstrual cycle, occasionally verified by ovarian hormone assessment, and aspects of anxiety ranging from vulnerability to panic (67) to response to treatment (68). Static assessments of ovarian hormones encompass most of the menstrual cycle and anxiety literature. In a review examining anxiety symptom fluctuations across the menstrual cycle, only 3/14 (21%) studies used more than two assessment points for the purposes of detecting hormonal changes (66). Those that used multiple assessment points did so weekly, thus, detecting overall trends in change but lacking in a comprehensive picture of fluctuations. Relatedly, in the nicotine reinforcement literature, in-lab paradigms examined acute associations between ovarian hormones and aspects of smoking including craving (69) and the effects of nicotine (55). Despite calls to include direct measurement of ovarian hormones in addiction research (44), a review of 11 studies on addictive behaviors across the menstrual cycle (70) identified only one smoking study that used an objective assessment of estradiol and progesterone. Specifically, Snively et al. (71) utilized repeated measurement of ovarian hormones (via plasma) during the menstrual cycle to confirm cycle phase status but changes in hormones were not considered. Frequent (repeated) assessment of hormones is important to understanding how *changes* in estradiol and progesterone levels may influence anxiety and nicotine craving.

#### Limitation 4: limited study of within-person variability

Existing work has largely focused on understanding between-person differences in both the anxiety and nicotine reinforcement literatures. Anxiety and craving are internal experiences that vary at both the between- and within-person level, are known to be influenced by person-specific factors, and are subject to recall biases when assessed retrospectively. Meaningful differences between anxiety and craving experiences are being missed through the stratification of menstrual cycle phases that are routinely grouped together in these literatures (25). This issue of overlooking variance is exacerbated by well-known person-specific factors affecting anxiety and nicotine reinforcement. Anxiety sensitivity and distress intolerance are two emotion-specific individual vulnerability factors that have been robustly linked to the risk and maintenance of anxiety and various aspects of smoking and nicotine reinforcement (72). These vulnerability factors, among others, may moderate the anxiety-smoking link and require within-person approaches to adequately address. Finally, use of within-person repeated momentary assessment allows for more valid reporting of anxiety and craving and mitigates reliance on retrospective reports (23). This “high resolution” assessment strategy provides critical information about the nature of both the ovarian hormone- anxiety and ovarian hormone- nicotine reinforcement links being elucidated. Thus, the study of intraindividual changes in anxiety and craving during the menstrual cycle will enhance our understanding of how and when hormonal fluctuations influence anxiety and craving during the menstrual cycle.

### Example protocol: Project SHE

To address the abovementioned limitations, we developed Project SHE (*Smoking*, *Hormones*, and *Emotion*), a National Institute on Drug Abuse (NIDA)-funded study that explored how fluctuating estradiol and progesterone during the menstrual cycle influence anxiety and cigarette craving in female smokers. We present the protocol as an example of how to advance methodological approaches in this research area.

### Participants

The sample was comprised of female smokers (*N* = 50; *M*_age_ = 32.4, SD = 5.3; 70.0% white race) who reported being daily combustible cigarette users for 14.9 (SD = 5.9) years and smoked an average of 12.2 (SD = 5.3) cigarettes per day in the past week. Participants were recruited through flyers on a university campus and in the surrounding area and through an online advertising agency which convened a sample representative of the broader population. Target sample size was determined to detect medium effect sizes. Participants were eligible for the study based on the following criteria: (a) between ages 18–40 years; (b) reported daily use of ≥5 cigarettes per day; (c) verification of smoking status via ≥10 ppm expired carbon monoxide (CO); (d) self-reported normal menstrual cycle (i.e., length of 25–35 days) that did not regularly vary in length month-to-month by ≥7 days. Exclusion criteria included: (a) current use of smoking cessation treatments; (b) pregnancy (determined by pregnancy test) or within 3-month postpartum; (c) breastfeeding within the last 3 months; (d) use of medications that influence ovarian hormone levels or the menstrual cycle [e.g., (25)], (e) history of medical conditions that effect the ovarian hormones/menstrual cycle (e.g., ovarian hypofunction, adrenal disorders), (f) social instability likely to hinder adherence to home-based study protocol (e.g., houselessness). Regarding psychiatric exclusion, we aimed to be as inclusive as possible to recruit a sample population that reflected “real world” smokers (per *Limitation 1*). Smokers with DSM-5 anxiety and related disorders (e.g., major depression, stable bipolar I/II, PTSD, OCD, PMDD) were allowed to participate in the study. We only excluded participants who presented with severe psychiatric instability, which we defined by: evidence of psychotic symptoms; current past-year unstable bipolar disorder; current past-year severe DSM-5 alcohol or substance use disorder; active suicidal or homicidal ideation; or current low-weight eating disorder (BMI < 17). These exclusionary criteria were selected to reduce potential for dangerous or protocol-interfering behaviors.

### Procedures

Participants completed an initial baseline visit via HIPAA compliant Microsoft Teams video call to determine eligibility during which the Mini International Neuropsychiatric Interview [M.I.N.I. version 7.0.2; (73)] and the PMDD module from the SCID-I (74) were administered to document the presence of DSM-5 disorders. The initial visit also included the collection of demographic information, measures of smoking history and cigarette dependence, and medical history, including a past-month confirmation of menstrual cycle length. An estimated first day of bleeding for the next cycle was calculated based on the beginning date of the last menstrual cycle as a reference and was used to approximate the current cycle day (e.g., if an individual’s last cycle started 10 days prior to the baseline assessment, they were considered to be on cycle day 10). Protocol initiation was not tied to cycle day or phase; natural variability in cycle start-date was designed to help to protect against order-effect biases that can occur in within-subject tests. Thus, participants were able to begin the study at any point in their menstrual cycle. Participants were oriented to the daily saliva and survey protocol and given an opportunity to practice saliva collection and momentary assessments, at which point participants were officially enrolled into the study monitoring period for one menstrual cycle. The exact length of the study monitoring period was dependent upon length of the menstrual cycle and was prospectively monitored by study staff. Menstrual status (presence of any bleeding; day of period) was assessed each evening during the study (75) and verbally confirmed at the weekly follow up appointments to ensure accuracy and accommodate for any missed reports.

#### Saliva collection protocol

To address *Limitations 2 and 3*, daily salivary sampling methods were utilized to objectively assess progesterone and estradiol levels every day during the cycle. Salivary sampling methods have an advantage over plasma methods in that the former is almost exclusively a measure of unbound (i.e., biologically available) hormone (76). Participants were introduced to saliva collection at the orientation visit, at which time they were provided with saliva collection kits (Salimetrics, LLC) and a portable freezer pack for transporting samples. Participants were instructed on using a saliva collection aid to facilitate whole saliva (passive drool) into the collection vial. Participants provided daily 1.8 mL samples at their home within 30 min of waking, labeled the vial with date/time of collection, stored it in the back of their home freezer, and transported back to the lab weekly. Frozen samples were stored in a − 80°C freezer until being sent for assay. Salimetrics 17*β*-Estradiol Enzyme and Progesterone Enzyme Immunoassay Kits (Salimetrics, State College, PA) were used. Saliva sample viability was determined by: (a) clear samples with no discoloration (e.g., blood, lipstick, yellowish tinge) or observable particle contaminants (e.g., food); (b) 1.8 mL of saliva; (c) no air bubbles; (d) properly labeled; and (e) frozen solid. To bolster compliance, a push-notification was sent to participants each morning reminding them to provide a saliva sample. They were prompted to complete a brief saliva report at the time of collection that included an uploaded photo of the collected saliva sample and to indicate whether there were any problems in providing the sample.

#### EMA protocol

**Figure 1:**
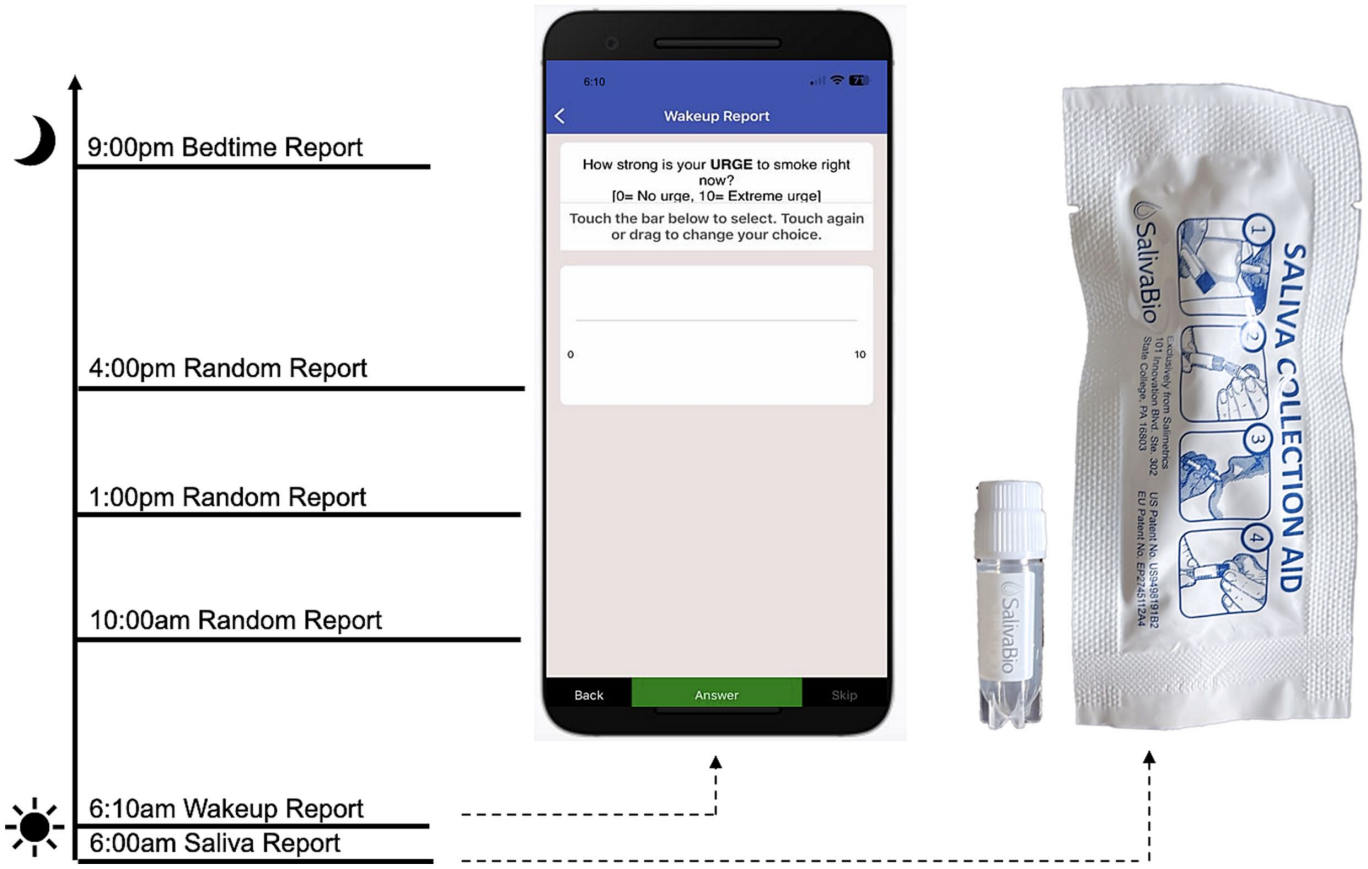
Hypothetical example of the daily protocol schedule.

To address *Limitation 4*, we used an ecological momentary assessment (EMA) protocol to capture within-person associations between ovarian hormones, anxiety, and craving throughout the menstrual cycle. The EMA protocol was delivered through MetricWire, a HIPAA compliant platform with a mobile application, that can deliver signal-contingent, time-contingent, and event-contingent reports. Participants downloaded the application to their smartphones during the orientation visit and were given an opportunity to practice navigating through the platform and completing surveys. In this study, participants received a push-notification each morning to complete a report, in addition to three random (signal-contingent) reports throughout the day, and one bedtime report each evening. Anxiety was assessed with a single item (*“How anxious do you feel right now? [0 = not at all, 10 = highest]”*). Cigarette craving was also assessed with a single item (“*How strong is your urge to smoke right now? [0 = No urge, 10 = Extreme urge]”*). In addition, morning reports included items that assess tobacco and substance use from the previous day (e.g.*, “How many cigarettes did you smoke yesterday?*”) to capture substance use quantity and frequency. A hypothetical protocol day including EMA and salivary assessments is depicted in Figure 1.

#### Protocol compliance

Compliance with the saliva collection and EMA methods was high. A total of 94.5% of saliva samples (*n* = 1,316) were returned, of which 94.2% (*n* = 1,240) were valid. The invalid samples (*n* = 76) were due to: thawed or discolored saliva (*n* = 36), out of range PH levels per assay results (*n* = 31), recent smoking/eating (*n* = 3), or insufficient amount of saliva to assay (*n* = 6). Regarding compliance with the EMA, 86.2% (*n* = 1,213) of morning reports were completed; 88.4% (*n* = 1,243) of at least one random report/day and 67.4% (*n* = 947) of at least two random reports/day were completed; and 82.3% (*N* = 1,159) of bedtime reports were completed.

### Illustrative case examples

**Figure 2:**
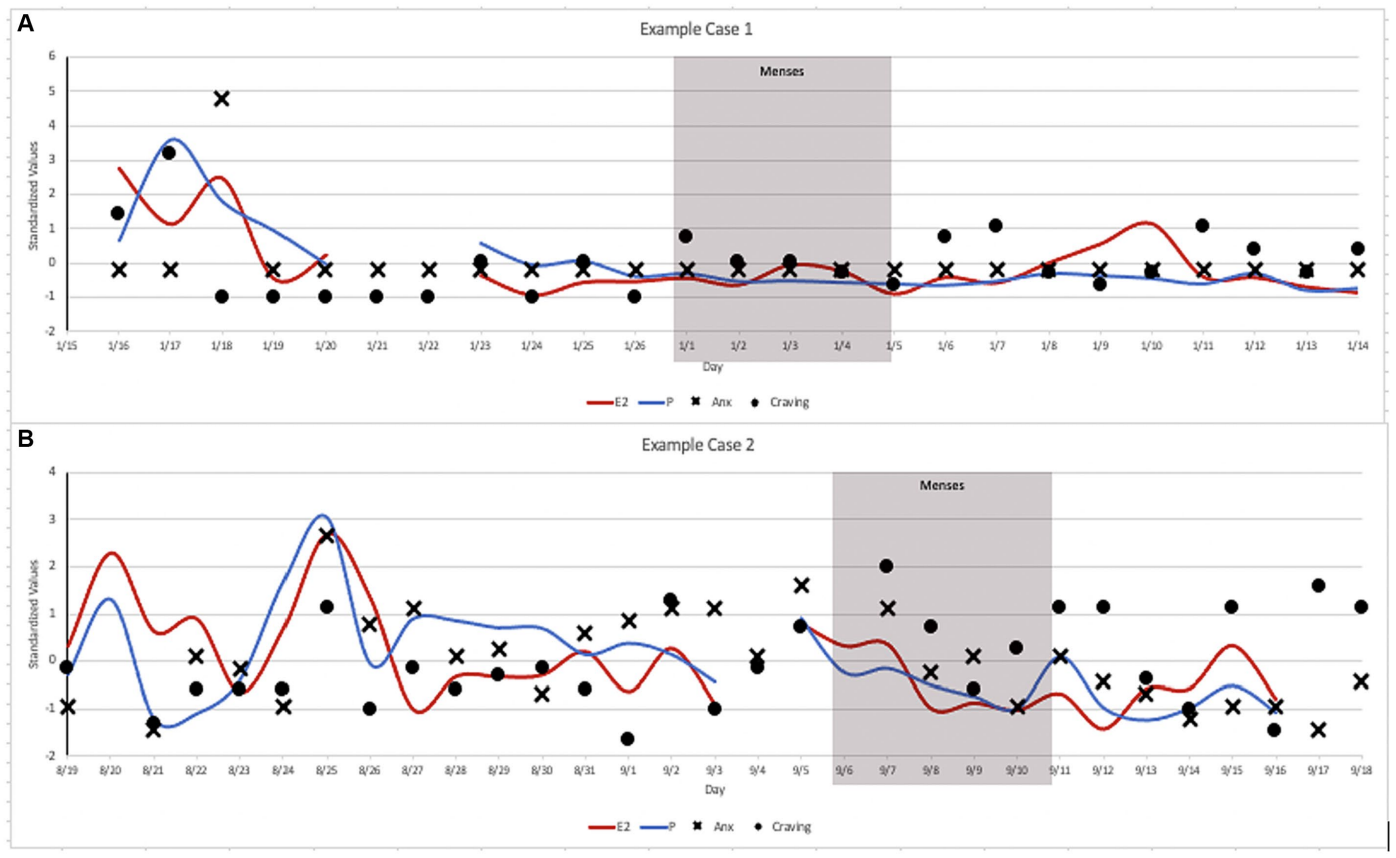
Case Illustrations: **(A)** Case 1 was a 26-year-old, White, female, who reported smoking 20 cigarettes/day with moderate cigarette dependence (FTCD = 5), with no history of psychopathology. **(B)** Case 2 was a 27-year-old, White, female, who reported smoking 20 cigarettes/day with moderate cigarette dependence (FTCD = 5), who had elevated high anxiety sensitivity (ASI-3 = 32) and met criteria for a major depressive disorder and a history of panic attacks.

We present two illustrative case examples to highlight the intra- and inter-individual fluctuations in anxiety and craving across the menstrual cycle (Figure 2). Data were transformed into standardized values for the purpose of illustration along a consistent axis. We present data from two participants—Case 1 with no history of psychopathology and Case 2 with anxiety and depression psychopathology—who were otherwise comparable in terms of demographics, cigarettes/day, and cigarette dependence. As seen in Figure 2, relatively limited variability/range in anxiety and craving were observed in Case 1 across the menstrual cycle. In contrast, ample fluctuation/variability in daily anxiety and craving across the menstrual cycle in Case 2. These data highlight the value of collecting daily data to capture anxiety and craving during the menstrual cycle, especially in females who have clinically-elevated anxiety and/or anxiety vulnerability.

## Conclusion

The protocol presented here highlights feasible means of addressing limitations in these disparate literatures to date, unifying them in the process. Our protocol represents a critical first step in increasing representation among smokers with co-occurring anxiety psychopathology. Although we attempted to address many existing methodological limitations of the literature, we acknowledge limitations of the Project SHE protocol. First, data were collected over the course of a single menstrual cycle, thus we are unable to explore inter-cycle heterogeneity. Second, the use of alcohol was reported on 8.7% of all days sampled (across 29 participants) and the use of cannabis was reported on 3.7% of all study days sampled (across 8 participants). Although relatively infrequent, we did not examine the influence of other substance use on cigarette craving. Lastly, we opted to not measure luteinizing hormone (LH) as an attempt to limit additional participant burden, precluding assessment of anovulatory cycles. LH assessment would enable indexing of ovulation timing and should be considered in future extensions of this work.

Continued work in this area is necessary to better understand the dynamic covariation in hormonal changes, anxiety, and negative reinforcement-precipitated craving and smoking over the course of female’s menstrual cycles. Given the significant prevalence of comorbid psychopathology among female smokers, future studies might specifically *recruit*, rather than *exclude*, individuals with a range of diagnoses, including anxiety, mood, and substance use disorders.

## Data availability statement

The raw data supporting the conclusions of this article will be made available by the authors, without undue reservation.

## Ethics statement

The studies involving humans were approved by Rutgers University Institutional Review Board. The studies were conducted in accordance with the local legislation and institutional requirements. The participants provided their written informed consent to participate in this study.

## Author contributions

SF: Conceptualization, Funding acquisition, Writing – original draft, Writing – review & editing. JS: Writing – original draft, Writing – review & editing. DS: Writing – original draft, Writing – review & editing. BA: Writing – original draft, Writing – review & editing. GL-M: Funding acquisition, Writing – review & editing. SD: Funding acquisition, Writing – review & editing. DW: Funding acquisition, Writing – review & editing. MS: Funding acquisition, Writing – review & editing. AA: Funding acquisition, Writing – review & editing.
